# Analysis of keratometric measurements in accordance with axial length in an aged population

**DOI:** 10.1038/s41598-022-08194-0

**Published:** 2022-03-08

**Authors:** Sung Uk Han, Soyoung Ryu, Hyunjean Jung, Hyunmin Ahn, Sangyeop Kim, Ikhyun Jun, Kyoung Yul Seo, Tae-im Kim

**Affiliations:** 1 Institute of Vision Research, Department of Ophthalmology, Yonsei University College of Medicine, Seoul, South Korea; 2 Corneal Dystrophy Research Institute, Department of Ophthalmology, Severance Hospital, Yonsei University, Seoul, Republic of Korea

**Keywords:** Anatomy, Diseases

## Abstract

To investigate keratometric measurements according to axial length in an aged population. Patients requiring cataract surgery with keratometric measurements from four different ophthalmic devices (autorefractor/keratometer, Scheimpflug imaging, corneal topography/ray-tracing aberrometry, and partial coherence interferometry) between January 2016 and March 2021 were reviewed retrospectively. Cases for which four ophthalmic devices were deployed in the same order a day were included in this investigation. The corneal curvature of the flattest and steepest meridian, mean corneal curvature, corneal astigmatism, steepest axis location, and axial length were evaluated. In total, 250 eyes (137 patients) were included in the analysis. A negative correlation was found between mean corneal curvature and axial length, with correlation coefficients of 0.587, 0.592, 0.588, 0.591, 0.588, and 0.562 for autorefractor/keratometer, Scheimpflug imaging, corneal topography/ray-tracing aberrometry, partial coherence interferometry, total corneal refractive power of Scheimpflug imaging, and simulated keratometry of corneal topography/ray-tracing aberrometry measurements, respectively. No statistically significant differences were found for corneal astigmatism according to axial length. In axial length group of less than 26.0 mm, negative correlation was found between axial length and mean frontal corneal curvature while no correlation was found between axial length and corneal astigmatism. All four ophthalmic devices showed good inter-device reliability for mean corneal curvature but not corneal astigmatism.

## Introduction

When considering the optical role, axial length is the primary determinant of refractive error^1^. And corneal power also plays an important role^2^. Keratometry determines the curvature of the corneal surface, which can be expressed in diopters or as the radius of the curvature. Keratometers, corneal topographers, and anterior segment tomographers can provide corneal measurements^3^. As different devices measure differently, there is no standard technique for measuring keratometry. Thus, repeatability, reproducibility, and validity of measurement devices should be evaluated for reliability. As corneal curvature influences the degree of refraction and high corneal astigmatism can cause a range of visual problems (such as visual blurring, glare, halo, and monocular diplopia), evaluations of both the corneal curvature itself and corneal astigmatism are important for understanding the refractive characteristics of the cornea.

As mentioned above, axial length is the most powerful determinant of refractive error and it is well known that myopia is the most prevalent refractive error worldwide^1^. Previous studies have suggested that structural changes resulting in myopia occur due to stretching in the periphery parallel to the visual axis, posterior pole elongation, and global expansion of the vitreous chamber^4–6^. Many studies have examined the relationship between myopia and corneal curvature with inconsistent findings. For example, a study conducted in China demonstrated a lack of statistically significant correlations between myopia and mean corneal curvature^7^, while another study in Canada demonstrated negative correlations between myopia and corneal curvature^8^. Another cross-sectional study suggested that eyes with higher levels of myopia had steeper central corneal curvatures and flatter peripheral corneal curvatures^9^.

In the current study, we analyzed corneal measurements using six different measurements obtained with four commonly used keratometric devices and axial length with partial coherence interferometry. We investigated inter-device keratometric agreement among the four devices and also examined the role of corneal refractive power and axial length in total eyeball refractive error within an aged population.

## Results

A total of 250 eyes (137 patients) were included in the analysis. The mean age of the participants was 72.5 ± 19.1 years. Table 1 presents participant demographic and medical data.

**Table 1:** Demographics of study populations.

Characteristics	No
Eye (right:left) (%)	140 (56%):110 (44%)
Age, years (mean ± SD)	72.5 ± 13.5
Sex (male:female) (%)	97 (38.8%):153 (61.2%)
Total mean axial length (mm)	23.87 ± 1.41
< 22.0: 22.0–26.0: ≤ 26.0 (%)	15 (6.0%): 213 (85.2%): 22 (8.8%)
**Subgroup mean axial length (mm)**	
< 22.0	21.47
22.0–26.0	23.72
≤ 26.0	26.96
Mean corneal curvature (anterior) (Diopter)	44.24 ± 1.65
Mean corneal stigmatism (anterior) (Diopter)	0.98 ± 0.73

Data are presented as the mean (mm) ± standard deviation (range).

**Figure 1:**
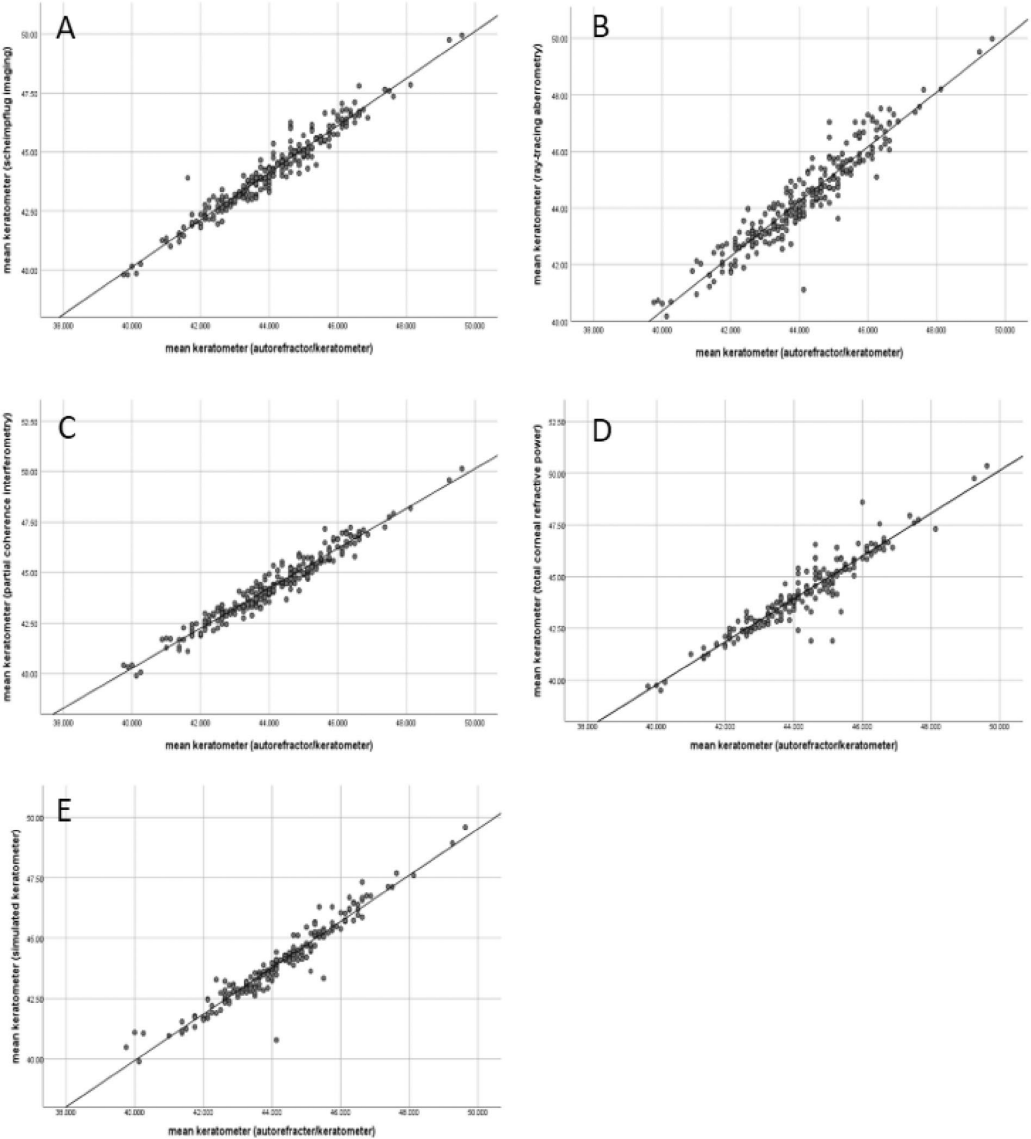
Bivariate analysis of mean keratometer of autorefracter/keratometer with others. (panel **A**) represents mean keratometer of scheimpflug imaging, (**B**) represents ray-tracing aberrometry, (**C**) represents partial coherence interferometry, (**D**) represents total corneal refractive power of scheimpflug imaging, and E represents simulated keratometer of ray-tracing aberrometry).

Mean corneal curvature measurements and astigmatism measured via four ophthalmic devices are presented in Table 2. The Kolmogorov–Smirnov test results demonstrate that the mean corneal curvature derived via the autorefractor/keratometer (ARK), Scheimpflug imaging system, ray-tracing aberrometry, and partial coherence interferometry measurements consistently showed a normal distribution (all *P* < 0.05). Figure 1 presents results for the bivariate analysis of the mean corneal curvature via ARK compared with that of the three other devices. Because of the extensive history of ARK as a standard keratometry method, we used this methodology as the referent. A very high correlation between mean corneal curvature in ARK with measurements conducted using the five other methods was observed; the highest correlation (0.974) was found with partial coherence interferometry.

**Table 2:** Mean corneal curvature and astigmatism measurement obtained using autorefractor/keratometer, scheimpflug imaging, corneal topography/ray-tracing aberrometry and partial coherence interferometry.

	Method I*	Method II^†^	Method III^‡^	Method IV^§^	Method V^Π^	Method V^#^
Flat corneal curvature	43.65 ± 1.65	43.75 ± 1.68	43.85 ± 1.65	43.77 ± 1.66	43.61 ± 1.84	43.52 ± 1.63
Steep corneal curvature	44.55 ± 1.68	44.69 ± 1.74	44.81 ± 1.75	44.87 ± 1.71	44.68 ± 1.81	44.45 ± 1.67
Mean corneal curvature	44.10 ± 1.63	44.22 ± 1.67	44.33 ± 1.66	44.32 ± 1.65	44.14 ± 1.78	43.99 ± 1.61
Corneal astigmatism	0.90 ± 0.68	0.94 ± 0.74	0.97 ± 0.70	1.10 ± 0.76	1.07 ± 0.81	0.93 ± 0.70

Data are presented as the mean (mm) ± standard deviation (range).

*Method I indicates autorefractor/keratometry.

^†^Method II indicates scheimpflug imaging.

^‡^Method III indicates corneal topography/ray-tracing aberrometry.

^§^Method IV indicates partial coherence interferometry.

^Π^Method V indicates total corneal refractive power of scheimpflug imaging.

^#^Method VI indicates indicates simulated keratometry of corneal topography/ray-tracing aberrometry.

**Figure 2:**
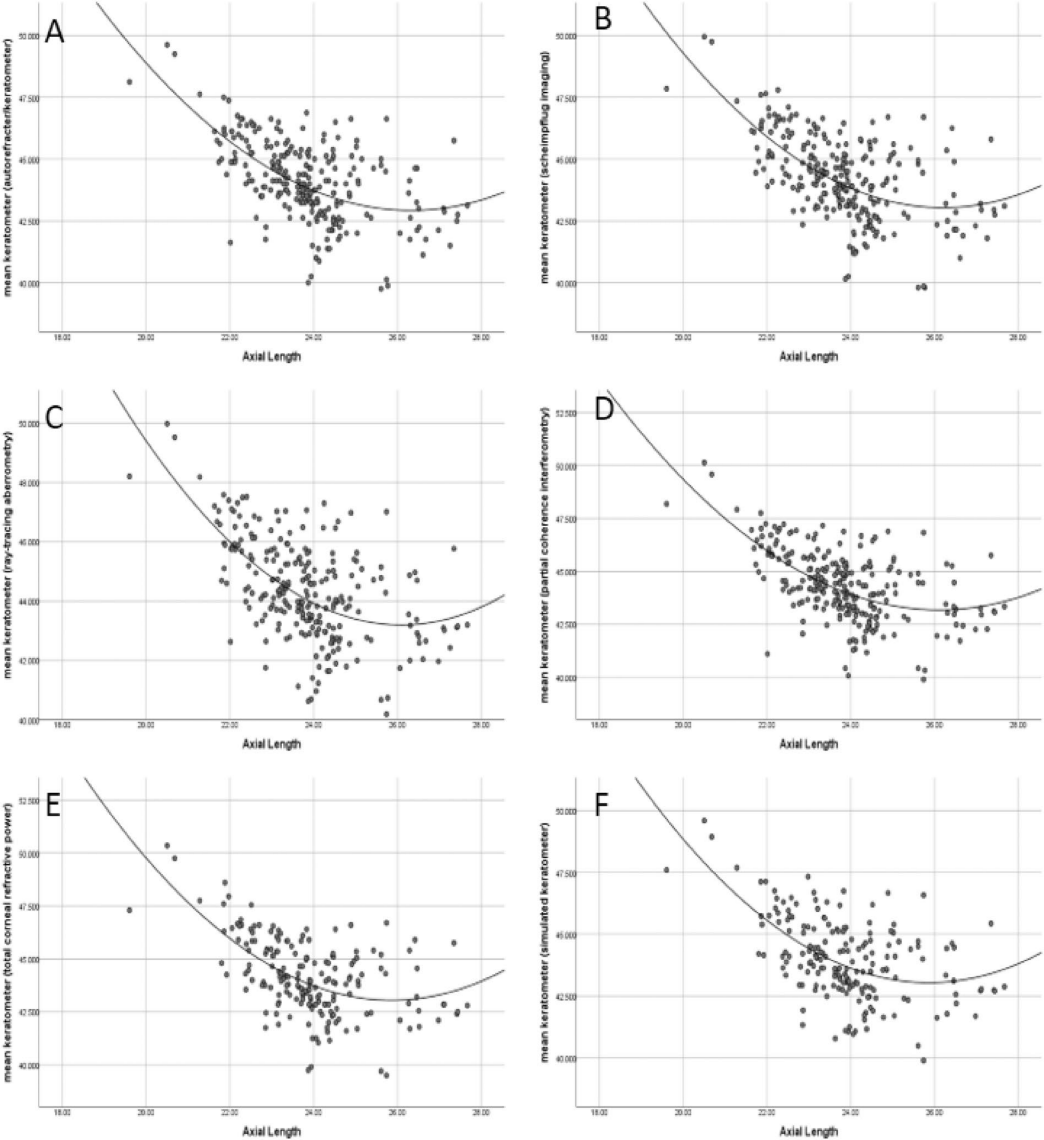
Polynomial regression analysis of mean keratometer of autorefracter/keratometer (panel **A**), scheimpflug imaging (panel **B**), ray-tracing aberrometry (panel **C**), partial coherence interferometry (panel **D**), total corneal refractive power of scheimpflug imaging (panel **E**), and simulated keratometer of ray-tracing aberrometry (panel **F**). Quadratic function of each panel were listed below. $y=0.11 {\mathrm{x}}^{2}-6.10x+125$ (**A**). $y=0.12 {\mathrm{x}}^{2}-6.39x+129$ (**B**). $y=0.13 {x}^{2}-6.78x+134$ (**C**). $y=0.12 {x}^{2}-6.69x+133$ (**D**). $y={0.20x}^{2}-10.1x+174$ (**E**). $y={0.17x}^{2}-8.72x+156$ (**F**).

**Figure 3:**
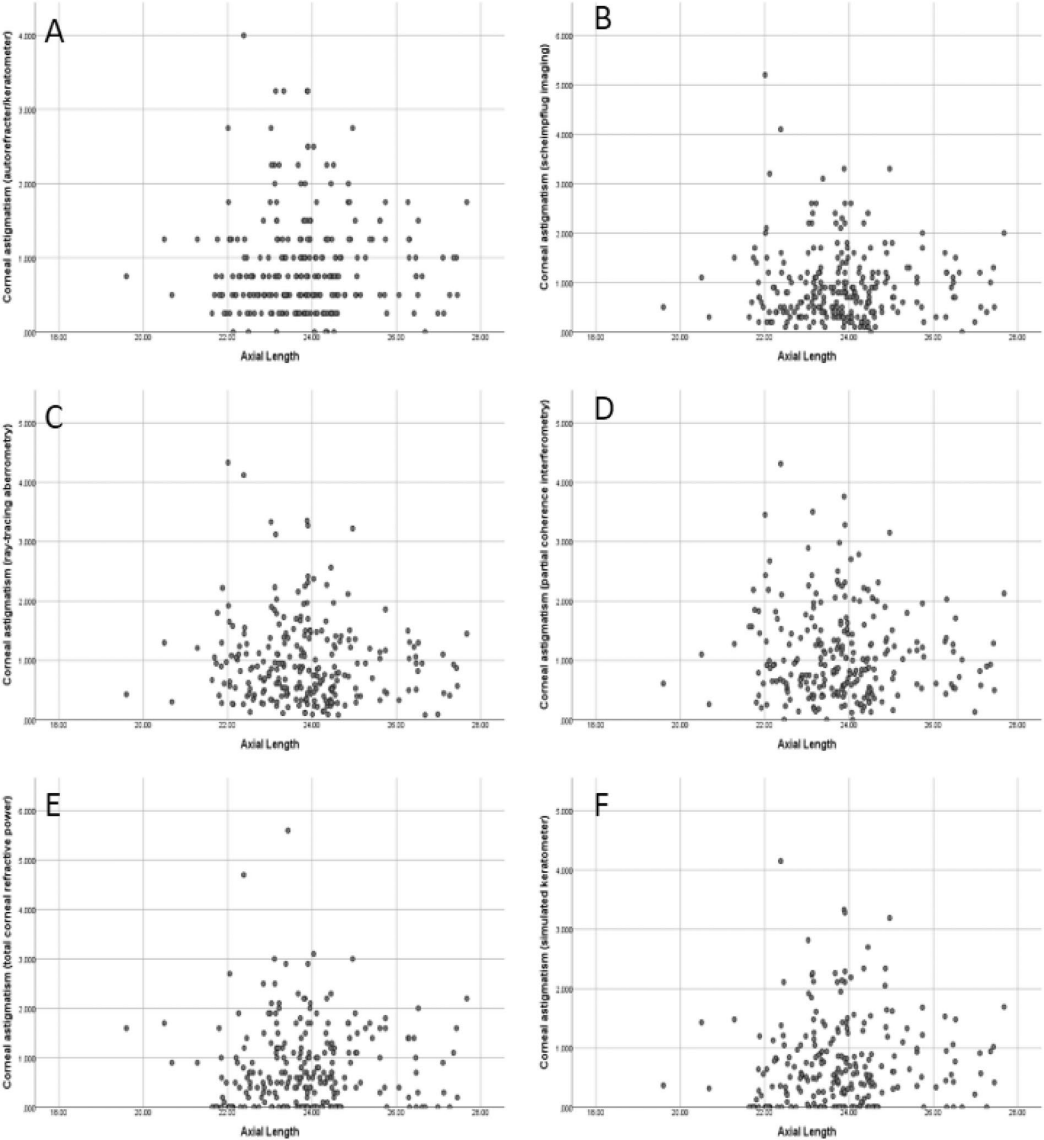
scatterplot of corneal astigmatism of autorefracter/keratometer (panel **A**), scheimpflug imaging (panel **B**), ray-tracing aberrometry (panel **C**), partial coherence interferometry (panel **D**), total corneal refractive power of scheimpflug imaging (panel **E**), and simulated keratometer of ray-tracing aberrometry (panel **F**).

Using polynomial regression analysis, mean corneal curvature and mean corneal astigmatism measurements in accordance with axial length were evaluated via the six methods (Figs. 2, 3). The correlation coefficients (CCs) for the mean corneal curvature according to axial length via the six methods ranged between − 0.562 and − 0.592 (− 0.587, − 0.592, − 0.588, − 0.591, − 0.588, and − 0.562, respectively), while corneal astigmatism showed no statistically significant correlations. These results indicate a moderate association (i.e., absolute values ranging between 0.5 and 0.7).

In Fig. 2, we included a quadratic function to evaluate the relationship between axial length and mean corneal curvature more intuitively. The x axes of symmetry on each of six graphs were 27.73, 26.63, 26.08, 27.88, 25.25, and 25.65 (the axis was longer than 26.0 mm in the first four and shorter than 26.0 mm in last two.) We also examined the relationship between axial length and corneal astigmatism. Unlike the mean corneal curvature, corneal astigmatism did not show any correlation with axial length.

Tables 3 and 4 present the comparability of the six different measurements with respect to mean corneal curvature and corneal astigmatism via CCs. All measurements of mean corneal curvature had high correlations with each other; ARK with partial coherence interferometry showed the highest correlation (0.974), whereas the lowest CC (0.929) was observed between the Scheimpflug imaging system and ray-tracing aberrometry measurements. Thus, in our study, ARK best matched with partial coherence interferometry in terms of mean corneal curvature. In a similar vein, when taking anterior corneal astigmatism into consideration, ARK with ray-tracing aberrometry showed the highest CC (0.820; Table 4).

**Table 3:** Correlation coefficient (CC) of 6 measurements regarding mean corneal curvature.

		Method I*	Method II^†^	Method III^‡^	Method IV^§^	Method V^Π^	Method VI^#^
Method I*	CC	1	0.971**	0.944**	0.974**	0.941**	0.962**
	*P* value		0.000	0.000	0.000	0.000	0.000
	N	250	250	250	250	188	188
Method II^†^	CC	0.971**	1	0.929**	0.959**	0.968**	0.947**
	*P* value	0.000		0.000	0.000	0.000	0.000
	N	250	250	250	250	188	188
Method III^‡^	CC	0.944**	0.929**	1	0.966**	0.922**	0.990**
	*P* value	0.000	0.000		0.000	0.000	0.000
	N	250	250	250	250	188	188
Method IV^§^	CC	0.974**	0.959**	0.966**	1	0.945**	0.961**
	*P* value	0.000	0.000	0.000		0.000	0.000
	N	250	250	250	250	188	188
Method V^Π^	CC	0.941**	0.968**	0.922**	0.945**	1	0.917**
	*P* value	0.000	0.000	0.000	0.000		0.000
	N	188	188	188	188	188	188
Method VI^#^	CC	0.962**	0.947**	0.990**	0.961**	0.917**	1
	*P* value	0.000	0.000	0.000	0.000	0.000	
	N	188	188	188	188	188	188

**Correlation is significant at 0.01 level.

*Method I indicates autorefractor/keratometry.

^†^Method II indicates scheimpflug imaging.

^‡^Method III indicates corneal topography/ray-tracing aberrometry.

^§^Method IV indicates partial coherence interferometry.

^Π^Method V indicates total corneal refractive power of scheimpflug imaging.

^#^Method VI indicates simulated keratometry of corneal topography/ray-tracing aberrometry.

*p*-value < 0.01 means statistically significant.

**Table 4:** Correlation coefficient (CC) of 6 measurements regarding corneal astigmatism.

		Method I*	Method II^†^	Method III^‡^	Method IV^§^	Method V^Π^	Method VI^#^
Method I*	CC	1	0.802**	0.820**	0.767**	0.660**	0.866**
	*P* value		0.000	0.000	0.000	0.000	0.000
	N	250	250	250	250	250	250
Method II^†^	CC	0.802**	1	0.817**	0.767**	0.734**	0.857**
	*P* value	0.000		0.000	0.000	0.000	0.000
	N	250	250	250	250	250	250
Method III^‡^	CC	0.820**	0.817**	1	0.783	0.612**	0.917**
	*P* value	0.000	0.000		0.000	0.000	0.000
	N	250	250	250	250	250	250
Method IV^§^	CC	0.767**	0.767**	0.783**	1	0.633**	0.869**
	*P* value	0.000	0.000	0.000		0.000	0.000
	N	250	250	250	250	250	250
Method V^Π^	CC	0.660**	0.734**	0.612**	0.633**	1	0.655**
	*P* value	0.000	0.000	0.000			0.000
	N	188	188	188	188		188
Method VI^#^	CC	0.866**	0.857**	0.917**	0.869**	0.655**	1
	*P* value	0.000	0.000	0.000	0.000	0.000	
	N	188	188	188	188	188	188

**Correlation is significant at 0.01 level.

*Method I indicates autorefractor/keratometry.

^†^Method II indicates scheimpflug imaging.

^‡^Method III indicates corneal topography/ray-tracing aberrometry.

^§^Method IV indicates partial coherence interferometry.

^Π^Method V indicates total corneal refractive power of scheimpflug imaging.

^#^Method VI indicates simulated keratometry of corneal topography/ray-tracing aberrometry.

*p*-value < 0.01 means statistically significant.

## Discussion

It is well known that hypermetropia occurs more frequently than myopia at birth^10^. As refractive components (including keratometric biometry and axial length) change with age, the current study aimed to evaluate correlations between axial length and keratometric biometry in an aged population. Anterior corneal curvature of the flattest and steepest meridian and the axis of these meridians were measured with four different ophthalmic devices. We first checked the inter-device repeatability of these biometric parameters and then evaluated associations with axial length. A previous study comparing Javal Schiotz type keratometry, IOL Master, TMS-4, and Pentacam HR measurements found that corneal curvature measurements showed high reproducibility and good correlation^11^. Comparisons of corneal curvature, astigmatism, and axis location in normal eyes obtained via ARK-700A and Atlas corneal topography also showed excellent repeatability and comparability^12^. Corneal curvature measured via the Scheimpflug imaging system likewise showed good reproducibility both anteriorly and posteriorly in a previous investigation^13^ In summary, there are many studies evaluating the comparability of corneal measurements via various instruments, and these studies have demonstrated varying results^14–16^.

We demonstrated the relevance of mean corneal curvature and astigmatism according to axial length via simultaneous ARK, Scheimpflug imaging system, ray-tracing aberrometry, and partial coherence interferometry evaluations. In a previous study, partial coherence interferometry showed higher astigmatism as compared with Scheimpflug imaging system, ray-tracing aberrometry, Atlas corneal topography, and Galilei dual Scheimpflug analyzer measurements^4,11^. In our study, we were able to compare each modality in order to evaluate mean corneal curvature and corneal astigmatism using bivariate correlation. We elicited similar results with respect to anterior corneal astigmatism measured by partial coherence interferometry, which showed statistically significant higher correlations as compared with other methods. An article comparing corneal astigmatism measured via an ARK 730A autokeratometer and simulated keratometry (simK) and total corneal power measured via a Galilei analyzer showed that astigmatism measured via total corneal refractive power (TCRP) tends to be higher than standard keratometric index-derived astigmatism^17^. Using a paired t-test method, we likewise found that total corneal astigmatism measured via TCRP was higher than that obtained through other methods, with the exception of partial coherence interferometry (which showed similar results that were at the level of statistical significance).

Another aim of this study was to evaluate keratometric measurements in accordance with axial length. Some previous studies have observed negative correlations between axial length and corneal curvature, with results similar to ours^2,18^. For example, one study by Olsen et al. using auto-refracto-keratometry (Nidek ARK 900, Nidek Co. Ltd, Gamagori, Japan) and keratometer and applanation ultrasound biometry (Nidek Echoscan US 800, Nidek Co. Ltd,) for axial length measurement revealed a negative correlation between corneal power and axial length with a CC of − 0.44^2^. Another study by Kinge et al. reported myopic shifts after puberty that were accompanied by vitreous chamber elongation (but not keratometry changes) among university students^19^. Another study likewise concluded that adult progression of myopia was related only to vitreous chamber elongation^20^. However, there have also been contradictory findings that show increasing axial length is related to steeper cornea^21–23^, such that van Alphen’s factor theory indicates a flatter cornea with longer axial length and Grosvenor’s stretch theory indicates a steeper cornea with longer axial length. Scott and Grosvenor explained this paradox by separating the normal growth of the eyeball from the abnormal growth that occurs during the development of myopia^23^. Moreover, another study reported that in low myopia or emmetropia groups, corneal flattening is caused by compensating mechanism of emmetropization whereas in moderate myopia, the cornea goes steep because of insufficient emmetropizing capacity^24^.

In our study, the *x* axes of symmetry on each of six graphs in Fig. 2 were 27.73, 26.63, 26.08, 27.88, 25.25, and 25.65. It means that, in axial length group of less than 26.0 mm, negative correlation was found between axial length and mean corneal curvature in all four measurements of anterior corneal curvature. However, the bivariate correlation analysis conducted to reveal the association between astigmatism and axial length did not find any statistically significant correlations between astigmatism and axial length within all four modalities (Fig. 3).

Though the definitions of short eye and long eye are not firmly fixed, when boundaries of 22.0 mm and 26.0 mm were assumed as the normal range for axial length, the mean corneal curvature showed negative correlations in accordance with axial length in our aged population with short and ordinary axial lengths. Previous animal experiments have found that the focal point of visual input causes axial length modulation and reduces refractive errors^25–27^, and further evidence indicates that this can occur similarly in human^1^. As mentioned in a previous study^24^, to a certain extent, it may be possible for the cornea to become flattened to adjust for refractive error. For example, previous research has observed that infants between the age of 3 and 9 months have the same basic pattern of eye lengthening, corneal flattening, and loss of refractive power as adults, showing similar trends to those observed in our study^1^.

An interesting reason why further studies are recommended is that our data were obtained from an aged population who were about to undergo cataract surgery; therefore, corneal astigmatism may have had crucial effect on intraocular lens (IOL) selection, especially in the selection of candidates for toric IOL. As shown in Table 3 and 4, the CCs of corneal astigmatism for each method were weaker than the CC of mean corneal refractive power. As the method we commonly used for selecting the IOL calculation was partial coherence interferometry, using method IV (partial coherence interferometry) as a guide, the CC of corneal astigmatism is between 0.633 and 0.869. Therefore, further research on more accurate methods for identifying toric IOL candidates according to axial length is needed. We considered the reason why CC of corneal astigmatism was lower than that of the mean corneal curvature. One possible reason is a difference in the minimum unit. In ARK, the minimum unit is 0.25, while that for other methods is 0.1. Another reason could be differences induced by the patient’s head tilting or chin sticking out, which can cause a vectorial difference. Other possible reasons include the zonal width difference of each method and the difference between the anterior and posterior keratometric axes, which cause corneal astigmatism differences between the anterior-only measurement and the total measurement.

This study had some limitations. First, when defining short and long eyes based on measurements of < 22.0 mm and > 26.0 mm, respectively, we evaluated a total of 15 short eyes and 22 long eyes in the current study. As this report aimed to examine correlations between axial length and corneal curvature, larger samples with both short eye and long eye would be preferable in future studies to confirm the correlations between these variables. Second, as the enrolled study participants visited the clinic for cataract surgery, the patient population was homogenously older. If we had included a more diversely aged population (with corresponding axial lengths), we may have achieved more conclusive and generalizable results. We recommend enrollment of a larger and more diverse study for future research.

In conclusion, we aimed to confirm the inter-device keratometric agreement of four ophthalmic devices and determine the relevance of corneal curvature (especially in mean keratometry and corneal astigmatism) according to axial length. We found that, in an aged population, keratometry via ARK, Scheimpflug imaging system, ray-tracing aberrometry, and partial coherence interferometry measurements using four different devices and six different methods presented good conformity in the mean corneal curvature but not in corneal astigmatism. Furthermore, the mean front corneal curvature showed a negative correlation with axial length in axial length group of less than 26.0 mm; however, no correlation was found between axial length and corneal astigmatism.

## Methods

### Participants

This study was approved by the institutional review board of Severance Hospital (IRB no. 1-2021-0045) and was conducted according to the tenets of the Declaration of Helsinki as well as Good Clinical Practices. As this study was designed as a retrospective study, the requirement for informed consent was waived. A total of 250 eyes (137 patients; 55 men and 82 women) requiring cataract surgery that were measured via four different ophthalmic devices between January 2016 and March 2021 were retrospectively enrolled in the current investigation. Participants who had no history of previous ocular surgery, trauma, or ophthalmic disease (with the exception of cataracts, myopia, hyperopia, and astigmatic ametropia) were selected. Exclusion criteria included severe ptosis, pathologic alteration of the cornea, retinal diseases involving macular degeneration, contact lens wearers, severe dry eye syndrome, and regular use of eye drops (other than artificial tears).

### Data acquisition

Anterior cornea curvature was examined via four devices evaluating ARK, Scheimpflug imaging, corneal topography/ray-tracing aberrometry, and partial coherence interferometry in 250 eyes. The flattest corneal curvature, steepest corneal curvature, mean corneal curvature, corneal astigmatism, and steep axis location were evaluated using four optical devices on the same day. The TCRP based on the Scheimpflug imaging system and simK via ray-tracing aberrometry were collected as well. The ARK (KR-7100; Topcon, Tokyo, Japan) uses six central spots of light around a central 3.0 mm zone to calculate the anterior curvature of the cornea; ARK is the most commonly used methodology for measuring keratometry. The Scheimpflug imaging system (Pentacam; OCULUS Optikgeräte GmbH, Wetzlar, Germany) acquires automatic measurements of the anterior and posterior corneal surfaces using a 360° rotating Scheimpflug camera^3^. All projected slits overlap at the central cornea to increase the accuracy of the central data, and anterior measurements within 3.0 mm of the cornea are used to calculate anterior corneal astigmatism^28^. Ray-tracing aberrometry (iTrace; Tracey Technologies, Corp., Houston, TX, USA) uses a placido disc videokeratoscope with a ray-tracing aberrometer^29^. Partial coherence interferometry (IOL Master 500; Carl Zeiss Meditec AG, Jena, Germany) measures anterior corneal astigmatism and curvature by analyzing the real position of six points within a hexagonal pattern reflection spot with a diameter ring of approximately 2.3–2.5 mm^28^.

TCRP data collected via the Scheimpflug imaging system and simK data collected via ray-tracing aberrometry were obtained for 188 eyes. The TCRP value was calculated via the refractive indices of air (1), the cornea (1.376), and aqueous humor (1.336) using Snell’s law, which does not require prior assumptions. We calculated TCRP for the central 4-mm zone of the pupil. The simK value was derived from the thin-lens formula and was calculated based on four points on a 3-mm circle centered on the corneal vertex^30^ SimK evaluations use the anterior surface to represent the total corneal power without knowing posterior corneal information^31^; this methodology uses the assumption that the anterior–posterior ratio is 0.822 and the corneal thickness is 500 μm^32^. Both TCRP via the Scheimpflug imaging system and simK via ray-tracing aberrometry can be used to calculate total corneal power. The sequence of measurement devices was the same in all cases. We performed the tests in the following order: ARK, Scheimpflug imaging system evaluations, ray-tracing aberrometry, and partial coherence interferometry. These test were performed sequentially for all cases. The axial length of the eyeball was measured using partial coherence interferometry. Mean corneal curvature indicates the middle value of the steepest and flattest corneal curvature and corneal astigmatism indicates the gap between the steepest and flattest corneal curvature. All four measurements were completed within 2 h with at least a 10 min interval between each test in a semi-dark room.

### Statistical analysis

Statistical analysis was performed using the Statistical Package for Social Sciences for Windows (version 15.0., SPSS, Inc., Chicago, IL, USA). Descriptive statistics are presented as means ± standard deviations. The normality of all data distributions was confirmed via the Kolmogorov–Smirnov test. Inter-device agreement was investigated using one-way analysis of variance, and the statistical significance of the inter-device correlation was analyzed via the Pearson correlation for the mean corneal curvature and corneal astigmatism. We evaluated correlations between anterior and total corneal curvature, corneal astigmatism, and axial length. *P* values < 0.05 were considered statistically significant.

## Data Availability

The data used to support the findings of this study, though not available in a public repository, will be available from the corresponding author upon request.
